# Large-scale identification of adverse drug reaction-related proteins through a random walk model

**DOI:** 10.1038/srep36325

**Published:** 2016-11-02

**Authors:** Xiaowen Chen, Hongbo Shi, Feng Yang, Lei Yang, Yingli Lv, Shuyuan Wang, Enyu Dai, Dianjun Sun, Wei Jiang

**Affiliations:** 1 College of Bioinformatics Science and Technology, Harbin Medical University, Harbin, 150081, China; 2 Center for Endemic Disease Control, Chinese Center for Disease Control and Prevention, Harbin Medical University, Harbin, 150081, China

## Abstract

Adverse drug reactions (ADRs) are responsible for drug failure in clinical trials and affect life quality of patients. The identification of ADRs during the early phases of drug development is an important task. Therefore, predicting potential protein targets eliciting ADRs is essential for understanding the pathogenesis of ADRs. In this study, we proposed a computational algorithm,Integrated Network for Protein-ADR relations (INPADR), to infer potential protein-ADR relations based on an integrated network. First, the integrated network was constructed by connecting the protein-protein interaction network and the ADR similarity network using known protein-ADR relations. Then, candidate protein-ADR relations were further prioritized by performing a random walk with restart on this integrated network. Leave-one-out cross validation was used to evaluate the ability of the INPADR. An AUC of 0.8486 was obtained, which was a significant improvement compared to previous methods. We also applied the INPADR to two ADRs to evaluate its accuracy. The results suggested that the INPADR is capable of finding novel protein-ADR relations. This study provides new insight to our understanding of ADRs. The predicted ADR-related proteins will provide a reference for preclinical safety pharmacology studies and facilitate the identification of ADRs during the early phases of drug development.

Adverse drug reactions (ADRs) are a major cause of drug failure in clinical trials, and also limit the use of effective drugs^1^. The early identification and prevention of ADRs have become an important issue for drug development. A principle of drug discovery is that the function of therapeutic targets is regulated to achieve the desirable therapeutic effects. However, drugs may also interact with off-targets to induce undesirable ADRs, which range from mild drowsiness to serious rhabdomyolysis. For example, terfenadine, a selective inhibitor of H1-receptors, is used to the treatment of allergies. However, terfenadine also causes arrhythmias due to the off-target inhibition of the human Ether-à-go-go-Related Gene (hERG)^2^. Thus, the key to avoiding ADRs is the investigation of the pathogenesis of ADRs, specifically, the identification of the protein targets responsible for ADRs.

Some computational methods have been proposed to identify ADR-related protein targets^3^,^4^,^5^,^6^,^7^. They are mainly based on establishing the associations between drug-target interaction data and the drugs’ ADRs. For example, Lounkine *et al.* screened for targets of marketed drugs from 73 targets that were included in Novartis *in vitro* safety panels. The predictions were validated using the chemical databases and Novartis *in vitro* assays. ADRs for three drugs were evaluated by constructing a drug-target-ADR network^3^. However, experimental tests of the interactions between drugs and thousands of proteins are very expensive. Yang and Pan used molecular docking methods to predict drug-target interactions^4^,^5^,^6^. However, molecular docking methods cannot be applied when the 3D structures of the target proteins are unknown^8^. These approaches have focused on relatively few ADRs. Later, Kuhn *et al.* used known drug-protein and drug-ADR relations to identify systematically overrepresented protein-ADR pairs through the enrichment analysis^7^. However, this method is dependent on the availability of drug-target interaction data. Molecular information for only 34% (1,428/4,192) ADRs could be obtained. Furthermore, Kuhn *et al.*’s work was unable to detect the causal proteins for approximately half of all the 1,428 investigated ADRs. In summary, the above-mentioned methods for ADR-related protein prediction have various limitations. Therefore, novel prediction methods are urgently needed to advance experimental research.

The goal of this study is to develop a new strategy for systematically predicting the relations between proteins and ADRs. Currently, many studies used similarity as a measure to investigate the relations between drugs, targets and ADRs^9^,^10^,^11^,^12^. For example, Campillos *et al.* used ADR similarity to infer drug targets, indicating that drugs that caused similar ADRs had similar protein binding profiles^9^. Hence, common drugs shared by two ADRs (also called co-occurrence drugs) can reflect the relations between these two ADRs and their associated proteins. Brouwers *et al.* investigated the contribution of the protein network neighborhood to ADR similarity between drugs^10^. They found that similar ADRs were caused by sharing of drug targets and neighbor drug targets in the network. Additionally, drug targets with similar pharmacological actions tended to interact with each other in a protein-protein interaction network^11^. These studies suggested that ADR similarity and protein-protein interaction network could be used to detect the relations between ADRs and proteins. Protein targets with interactions in protein network tend to be related to similar ADRs. Based on such findings, a computational algorithm, Integrated Network for Protein-ADR relations (INPADR), was developed to infer potential relations between proteins and ADRs. First, the co-occurrence drugs were used to quantify the similarity between ADRs, and an integrated network was constructed by combining the protein-protein network, the ADR-ADR similarity network and the protein-ADR network. Then, the random walk was implemented on the integrated network to rank the candidate proteins for an ADR of interest according to the stable probability of the walker. Leave-one-out cross validation was used to evaluate the ADR-related protein prediction performance. An AUC of 0.8486 was obtained, which suggested that the INPADR is superior to previous methods and capable of predicting ADR-related proteins. Case studies of two ADRs further revealed the high performance of our algorithm. This study provides a practical method to detect ADR-related proteins and will be valuable for ADR screening in clinical drug-discovery trials.

## Methods

### Datasets

ADR-drug relations were extracted from the SIDER 2 database^13^, which includes data for 996 drugs implicated in 4,192 different ADRs labeled with a Unified Medical Language System (UMLS) Concept Unique Identifier (CUI). Protein target set was derived from public targetable protein databases^14^,^15^,^16^,^17^ and the literature^7^. The identifier for each protein was mapped to an Ensemble ID. After filtering redundant annotations across the databases, we finally obtained 3,198 unique proteins for further analysis.

### An integrated protein-ADR network

**Figure 1:**
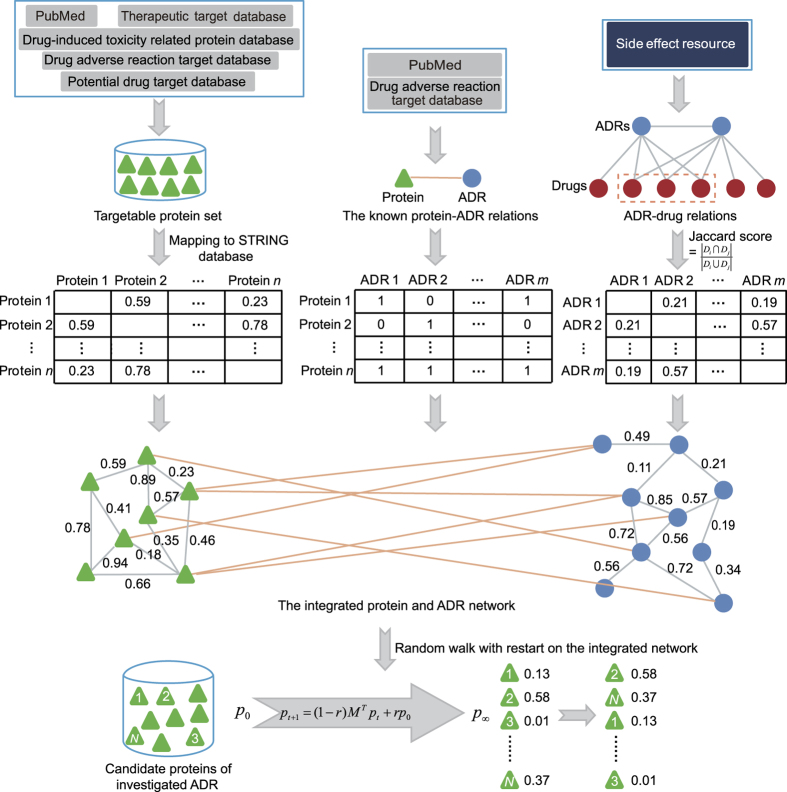
Workflow of the INPADR for predicting protein-ADR (Adverse Drug Reaction) relations. First, the protein interaction network is obtained by mapping a targetable protein set into the STRING database. The ADR similarity network is constructed using the co-occurrence of drugs. Then, the integrated protein and ADR network is constructed by connecting the protein interaction network and the ADR similarity network using known protein-ADR relations retrieved from Kuhn’s work and the DART database. Finally, random walk with restart on the integrated network is performed to obtain the stable probability, and candidate ADR-related proteins are ranked according to the stable probability.

An integrated protein-ADR network was constructed by combining three types of networks, namely the protein-protein interaction network, the ADR similarity network, and the protein-ADR network (see Fig. 1). Here, protein-protein interactions (PPI) were obtained from STRING v10^18^. STRING is a comprehensive database containing protein interactions from experimental evidence and comparative genomics prediction methods. It computed a confidence score for each protein interaction based on benchmarking the performance of the predictions against manually curated functional associations in the KEGG database, reflecting the confidence of the predicted interactions. After inputting the above-mentioned set of 3,198 targetable proteins, we obtained the PPI network including 73,833 interactions between 1,153 proteins. In the PPI network, the vertex set of *n* proteins is denoted as the set *VP* = {*vp*_1_, …, *vp*_*n*_}. Vertex *vp*_i_ and *vp*_j_ are linked by an edge, if the confidence score between *vp*_i_ and *vp*_j_ from STRING is more than 0. The confidence score was used as the weight of this edge. Next, we used the co-occurrence of drugs to evaluate ADR similarity. For two ADRs *i* and *j*, the Jaccard score was calculated as follows:


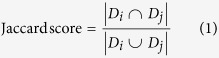


where *D*_i_ and *D*_j_ denote drug sets that cause ADR *i* and ADR *j*, respectively. The ADR similarity network was constructed according to the Jaccard score, where the set *VA* = {*va*_1_, …, *va*_m_} represents the vertex set of *m* ADRs; vertex *va*_i_ and *va*_j_ are linked by an edge, if the Jaccard score for them is greater than 0. The Jaccard score was used as the weight of this edge. Then, human protein-ADR relations were obtained from Kuhn’s work and the drug adverse reaction target database (DART, http://xin.cz3.nus.edu.sg/group/drt/dart.asp)^7^,^14^. DART is a manually curated database of the known target proteins related to the ADRs that are obtained from the literature. After preprocessing and verifying the names of proteins and ADRs, 503 relations between 245 proteins and 166 ADRs were used as the gold-standard dataset for evaluating the performance of the methods. Here, the identifiers for proteins were Ensemble IDs, and the ADRs were labeled with CUIs. The known 503 protein-ADR relations were represented as a bipartite graph, called the protein-ADR network. In the protein-ADR network, vertex *vp*_i_ and vertex *va*_j_ are connected by an edge if the protein *i* is related to the ADR *j*. Finally, we constructed the integrated protein and ADR network by connecting the PPI network and the ADR similarity network using the protein-ADR network.

### ADR-related protein prediction based on the integrated network

In this work, we used the random walk with restart-based predictor INPADR to infer potential protein-ADR relations (see Fig. 1). Random walk with restart (RWR) is a useful method to measure the proximity between two nodes of a network, and has been successfully applied to identify gene-disease relations and drug-target interactions^19^,^20^. The RWR method simulates a random walker starting from a source node (or a source set of nodes). At each step, the walker moves to one of the immediate neighbors with equal probability, and restarts from source nodes with a certain probability. After many iterations, the probability of finding the walker at node *x* converges to the stable probability, which is the proximity score. Therefore, the INPADR includes five steps as follows: (1) constructing the integrated network; (2) determining the initial probability; (3) determining the transition matrix; (4) performing RWR on the integrated network; and (5) obtaining the stable probability and ranking the candidate proteins for each ADR.

As mentioned above, the integrated network was generated by combining the PPI network, the ADR similarity network and the protein-ADR network. Let the matrix 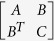 represent the adjacency matrix of the integrated network, where *A*_*n*×*n*_, *B*_*n*×*m*_ and *C*_*m*×*m*_ denote the adjacency matrices for the PPI network, the protein-ADR network and the ADR similarity network, respectively. *B*^T^ is the transpose matrix of *B*. Second, for an ADR *i* of interest, ADR *va*_i_ is considered as the seed node in the ADR similarity network. If protein *j* is related to ADR *i*, then *vp*_j_ is also considered as the seed node in the PPI network^21^. Let *u*_0_ denote the initial probability of the PPI network, where the seed nodes have the equal probabilities with the sum equal to 1 and the probabilities of non-seed nodes are zero. Therefore, the probability for a random walker of starting from each seed node is equal. Let *v*_0_ denote the initial probability of the ADR similarity network, where the probability of node *va*_i_ is 1 and the probabilities of other nodes are equal to 0. Therefore, the initial probability of the integrated protein and ADR network is 
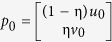
. The parameter 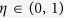 is used to measure the importance of the PPI network and the ADR similarity network. The RWR algorithm identified potential proteins related to ADR *i* by simulating a random walker’s transition from its current nodes randomly to neighbors in the integrated protein and ADR network starting at the given seed nodes. From Fig. 1, we can observe that not all the proteins are linked to ADRs. When the random walker is in the PPI network, it can jump to the ADR similarity network or stay in the PPI network. If it is at a protein node linked to ADRs, it can jump to one of the linked ADR nodes with the probability λ, or jump to other protein nodes with the probability 1−λ. Otherwise, it only moves in the PPI network. Here, the parameter λ is called the jumping probability of the random walker from the PPI network to the ADR similarity network or vice versa.

Third, the transition matrix needs to be defined to perform the random walk. Let 
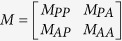
 denote the transition matrix of the integrated protein and ADR network, where *M*_*PP*_ and *M*_*AA*_ are intra-subnetwork transition matrices showing the probability from one protein (ADR) to other protein (ADR) in the random walk, respectively; *M*_*PA*_ and *M*_*AP*_ are inter-subnetwork transition matrices from the PPI (ADR similarity) network to ADR similarity (the PPI) network, respectively. The transition matrix *M* is defined as follows:

In the PPI network, the transition probability from vertex *vp*_i_ to *vp*_j_ is defined as


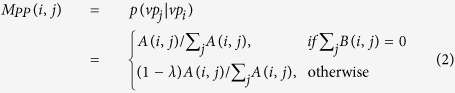


In the ADR similarity network, the transition probability from vertex *va*_i_ to *va*_j_ is defined as


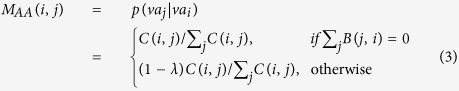


The transition probability from *vp*_i_ in the PPI network to *va*_j_ in the ADR similarity network is defined as


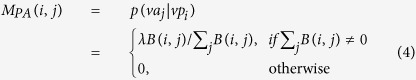


The transition probability from *va*_i_ in the ADR similarity network to *vp*_j_ in the PPI network is defined as


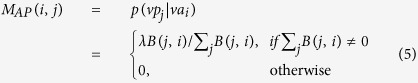


Next, a random walk is performed on the integrated protein and ADR network using the following Eq. (6)


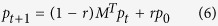


Here, *p*_t_ is a vector in which the *i*-th element is the probability of finding the random walker at node *i* at step *t*. The parameter 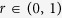 is the restart probability of the random walker at the seed nodes at each step. Finally, the stable probability 
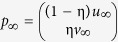
 is obtained after a number of iteration steps, and proteins are ranked according to 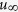. Based upon the previous work^19^, let the parameters *r*, λ and η equal to 0.7, 0.5 and 0.5 in our INPADR method, respectively.

## Results

### The basic properties of the protein-ADR network

**Figure 2:**
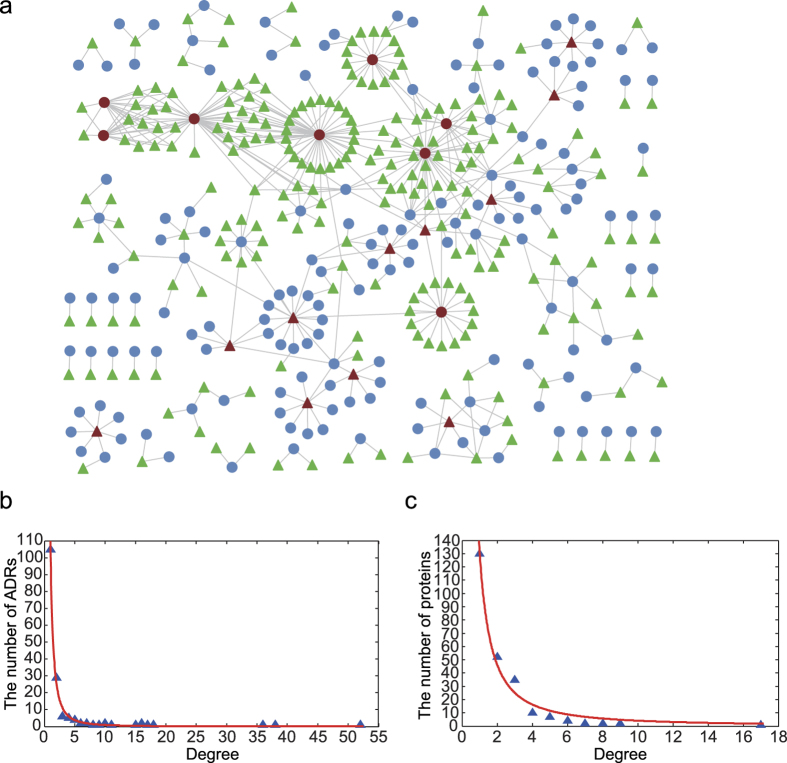
Visualization and characteristics of the protein-ADR network. (**a**) The protein-ADR network. If one protein was related to one ADR, they were linked by an edge. The protein and ADR nodes were shown by green triangles and blue circles, respectively. We colored the hub nodes red. The hub nodes were defined as the top 5 percent of proteins and ADRs with the highest degree. (**b**,**c**) Degree distribution of the ADR and protein nodes, respectively.

In this study, we first focused on the collection of known protein-ADR relations. We obtained 503 relations between 245 proteins and 166 ADRs (see Methods for details). The protein-ADR network was then constructed, which is shown in Fig. 2a. To view the global properties of the protein-ADR network, we performed network topology analysis (see Table 1). We further calculated the degree of ADR (protein) nodes, which is the number of proteins (ADRs) associated with the investigated ADR (protein). Each ADR interacted on average with 3 proteins, and each protein interacted on average with 2 ADRs. This result suggested that the dysfunction of multiple proteins contributed to the pathogenesis of ADRs. The degree distribution of the ADR and protein nodes both followed the power law distribution approximately with slopes of −2.059 and −1.508 and R^2^ = 0.9954 and 0.9824, respectively (see Fig. 2b,c).

### Evaluating ADR-related protein prediction using leave-one-out cross validation

The goal of our study is the prediction of novel protein-ADR relations based on an integrated network (see Methods for details). To evaluate the performance ability of the INPADR, leave-one-out cross validation (LOOCV) was applied to the 503 known protein-ADR relations. The algorithm cannot prioritize all protein-ADR relations for all the ADRs at the same time. Namely, it can only prioritize candidate proteins for an ADR. Hence, each known protein-ADR relation was removed from the integrated network and was taken as the test dataset in each cross-validation run. The investigated ADR and the remaining proteins related to this ADR were used as seed nodes. For the investigated ADR, all the proteins without known relations with this ADR were regarded as the candidate proteins. When one known protein-ADR relation was taken as a test dataset, we measured how well this test protein ranked relative to the candidate proteins of this ADR. If the ranking of the test protein exceeded a given threshold, then this protein-ADR relation was considered to be successfully predicted by the INPADR.

When LOOCV was performed, the sensitivity and specificity for each threshold were calculated. The sensitivity indicates the percentage of the test proteins whose rank is higher than a given threshold, namely the ratio of successfully predicted protein-ADR relations to the total known protein-ADR relations. The specificity indicates the percentage of proteins that are below the threshold. The receiver operating characteristic (ROC) curve plots the sensitivity versus 1-specificity at different thresholds. Then, the area under the ROC curve (AUC) was calculated, which was used as a measure to evaluate the algorithm performance.

**Figure 3:**
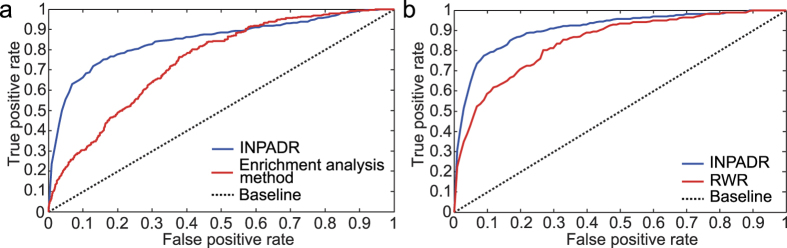
Method evaluation. (**a**) ROC curve of the INPADR and enrichment analysis method. The INPADR and enrichment analysis method are compared in terms of ROC curve and AUC using leave-one-out cross validation of 503 known protein-ADR relations. (**b**) ROC curve of the INPADR and the RWR. In the process of leave-one-out cross validation, the RWR only on the protein interaction network just is evaluated on the ADRs related with at least two proteins. Here, 398 known ADR-protein relations for ADRs related with at least two proteins are considered as the gold-standard set.

Here, we compared our INPADR with the enrichment analysis method based on a hypergeometric test using the integrated protein and ADR network. Enrichment analysis based on a hypergeometric test or Fisher’s exact test is a typical method for measuring the significance of the associations between two variables^7^,^22^,^23^,^24^. Kuhn *et al.* used Fisher’s exact test to identify associations between ADRs and proteins by integrating drug-ADR relations and drug-target interactions^7^. Jiang *et al.* used the hypergeometric distribution to prioritize the entire microRNAome for diseases based on a heterogeneous disease and miRNA network^22^. After performing LOOCV, the INPADR yielded an AUC of 0.8486, while the hypergeometric distribution method only produced an AUC of 0.7437 (Fig. 3a). This result suggested the INPADR can recover known, experimentally verified protein-ADR relations, and has the power to predict potential protein-ADR relations.

### Parameter effect of the INPADR

**Figure 4:**
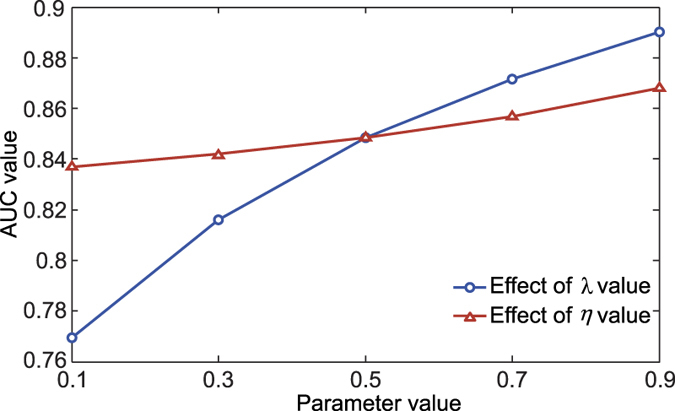
The effects of two parameters on the cross-validation results of the INPADR. The AUC values were calculated for different values of one parameter and the other parameter was fixed at 0.5.

In the INPADR, there were three parameters: restart probability *r*, jumping probability λ, and η which controls the impact of two types of seed nodes. It has been shown that the restart probability has little effect on the predictive result^19^,^20^,^21^. Therefore, we chose a restart probability of 0.7 according to a previous study^19^. The jumping probability λ is responsible for the reinforcement between the PPI network and the ADR similarity network. If λ = 0, the random walker will move in only one type of subnetwork. If λ = 1, the random walker will move only in the protein-ADR network. Namely, he will not reach nodes only in the PPI network or the ADR similarity network. If λ is larger, there is greater mutual dependence of ranking between proteins and ADRs. To investigate the effect of the parameter λ, we set various values of λ ranging from 0.1 to 0.9. The performance of the INPADR was measured using the AUC in the process of LOOCV. The result is shown in Fig. 4. The performance was improved with an increase of the λ value. When the λ value ranged from 0.5 to 0.9, the AUC values were distributed from 0.85 to 0.89. The performance was comparatively poor for λ values <0.5, but was superior to the hypergeometric distribution method. It suggested that the INPADR could successfully control the reinforcement between the PPI network and the ADR similarity network.

The parameter η regulates the impact of two types of seed nodes, namely, the protein node and ADR node. If *η* = 0.5, then the two subnetworks hold the same importance. If *η* is greater than 0.5, the random walker tends to return to the seed ADRs. The effect of the parameter value on cross-validation results according to the AUC is shown in Fig. 4. The performance of the INPADR slightly improved when η was set above 0.5, suggesting that the ADR similarity network has an important function for prioritizing the ADR-related proteins.

### Comparison with similar method

To further emphasize the importance of the ADR similarity network for the prediction of protein-ADR relations, we compared the INPADR with the RWR (Random Walk with Restart) on only the PPI network^21^. The RWR method is an iterative walker’s transition from the current node to its neighboring nodes starting at given seed nodes. Therefore, in the process of LOOCV, the RWR needs at least two proteins for an ADR due to performing a random walk only on the PPI network. Hence, in this comparative analysis, only ADRs related with at least two proteins were considered. We obtained 398 protein-ADR relations between 218 proteins and 61 ADRs as gold-standard data. We then performed LOOCV for each ADR. As shown in Fig. 3b, the ROC curve of the INPADR is above the RWR. The INPADR obtained an AUC value of 0.908, which is higher than the AUC value for the RWR (0.8437). Therefore, the INPADR is superior to the RWR on the protein network alone.

### Potential ADR-related protein prediction and case study

After confirming the power of the INPADR to predict novel ADR-related proteins, we applied the INPADR to predict potential proteins for each ADR investigated in this study. The INPADR is a top*-k* proximity query method which obtained the *k* proteins with the highest proximity (i.e., the highest stable probability) from a given ADR in the integrated network. In our study, we chose the *k* of 50 according to previous studies^25^,^26^. We publicly released the predictive results for each ADR in Supplementary Table S1, which will provide an experimental reference for biologists. In addition, we performed case studies of two ADRs (hypertension and atherosclerosis) to show the application of the INPADR in predicting the potential ADR-related proteins. For each ADR, all the known ADR-related proteins were used as seed nodes, and candidate proteins (non-seed proteins) were prioritized by the INPADR. The top 50 predictive proteins for two ADRs (see Supplementary Table S1) were validated by literature mining and the DisGeNet platform^27^. Hypertension is defined as persistent high systemic arterial blood pressure. There were 18 known proteins related to hypertension. Among the top 50 predictive hypertension-related proteins, 39 proteins had been confirmed to be associated with hypertension by experimental evidence in the literature. Atherosclerosis sometimes occurs in patients treated with ropinirole and naltrexone. There were 7 known proteins related to atherosclerosis. Among the top 50 predictive atherosclerosis-related proteins, 46 proteins had been confirmed to be associated with atherosclerosis by experimental evidence in the literature.

**Figure 5:**
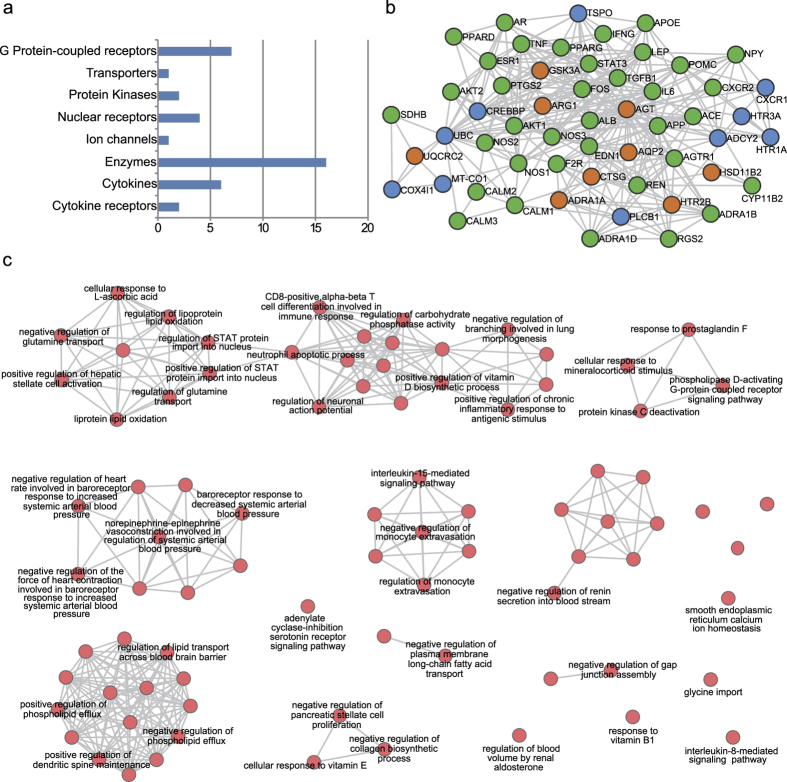
The biological characteristics of the potential proteins related to hypertension. (**a**) KEGG classification of the top 50 potential proteins related to hypertension. (**b**) The interactions between known hypertension-related proteins and the top 50 potential proteins. Orange nodes denote known seed hypertension-related proteins. Green nodes denote validated top 50 potential proteins. Blue nodes denote unvalidated top 50 potential proteins. (**c**) A similar network of enriched GO biological processes by the top 50 potential proteins is built by the ClueGo based on the common genes between different GO terms. The biological processes are labeled, which are confirmed in the literature.

A list of the top 50 potential hypertension-related proteins corresponded to 49 individual genes. According to KEGG gene classification, GPCR receptors and enzymes were the two largest classes of potential hypertension-related proteins (see Fig. 5a). GPCR receptors (23%) and enzymes (50%) are the most important classes of target proteins in the human genome^28^. The classification of top 50 potential atherosclerosis-related proteins is shown in Supplementary Fig. S1. Enzymes were also the largest class of potential atherosclerosis-related proteins.

Additionally, the interactions between known ADR-related proteins and the top 50 potential proteins were shown for hypertension and atherosclerosis according to the protein-protein confidence scores (Fig. 5b: hypertension; Supplementary Fig. S1: atherosclerosis). If the confidence score between two proteins was equal to or larger than 0.7, then the edge linking them was retained. It was found that ADR-related proteins preferred to form some modules, which was consistent with the basic assumption of the INPADR.

Finally, to verify the rationality of the INPADR, we performed functional enrichment analysis for the potential ADR-related proteins using the ClueGo Cytoscape plug-in^29^. Meanwhile, ClueGo constructed a GO biological process similarity network based on shared genes between Go terms (see Fig. 5c: hypertension; Supplementary Fig. S1: atherosclerosis). A review of the literature revealed that half of the enriched biological processes were related to hypertension (The biological processes validated in the literature for hypertension and atherosclerosis are listed in Supplementary Table S2). For instance, STAT (signal transducers and activators of transcription) proteins are a family of transcription factors that are activated by phosphorylation. Once phosphorylated, STAT protein dimers are transported to the nucleus. STAT family members were involved in diverse biological functions including cell differentiation, proliferation, development, apoptosis, and inflammation^30^. In particular, it was reported that STAT3 played an important role in hypertension^31^. Thus, changes in STAT protein import into the nucleus can lead to the progression of hypertension^32^. The renin-angiotensin system (RAS) is a hormonal cascade regulating blood pressure. Inappropriate activation of the intrarenal RAS was a critical factor to the pathogenesis of hypertension. The plasma renin concentration was used to measure the overall activity of the RAS^33^. Inhibition of renin release into the bloodstream was associated with hypertension. Protein kinase C (PKC) is a family of phospholipid-dependent serine/threonine kinases distributed in different blood vessels. Multiple factors lead to hypertension including changes in neuronal, hormonal, renal and vascular control mechanisms of blood pressure^34^. The activity of PKC can perturb one or more of these physiological control mechanisms, causing persistent increases in blood pressure and hypertension^35^. Hence, dysfunction of protein kinase C deactivation could be associated with hypertension. Therefore, the independent case studies further showed the reliability of our INPADR for identifying the protein-ADR relations.

## Discussion

The identification of ADR-related proteins can be used to explain the molecular mechanisms of reported drug-ADR pairs and can be helpful for *in vitro* ADR assessment at an early phase of drug development. In this study, based on the hypothesis that similar ADRs are caused by proteins that interacted with each other in the PPI network, a computational predictor, INPADR, was developed to identify the potential protein-ADR relations. We first constructed the protein-ADR network based on experimentally verified protein-ADR relations. Network topological analysis revealed that the degree of distribution of the ADR and protein nodes in the protein-ADR network approximately followed the power law distribution. Similar to other biological networks, the protein-ADR network was a scale-free network. We then constructed an integrated network by connecting the PPI network and the ADR similarity network using the protein-ADR network. Then, the candidate proteins for each ADR were ranked by implementing a random walk with restart on the integrated network using the ADR and corresponding known ADR-related proteins as seed nodes. LOOCV was used to evaluate the performance of the INPADR. The results revealed that the INPADR was superior to the hypergeometric test and the RWR using only the protein interaction network. The INPADR had the ability to recover the known experimentally verified ADR-related proteins. Furthermore, we performed case studies for two ADRs, and most of the predictive results can be confirmed by database and literature. These case studies further demonstrated that the INPADR has good performance and application value for prioritizing candidate ADR-related proteins. Overall, the good performance of the INPADR was attributed to the fact that the INPADR integrated three different networks into a heterogeneous network, and the experimentally verified ADR-protein relations were used as a gold-standard dataset in cross-validation and seed set to identify potential ADR-related proteins. In addition, the INPADR is based on the topological structure of the integrated network. The scarcity of the experimentally verified protein-ADR relations leads to the incompleteness of the integrated network. Obtaining more known ADR-related proteins will improve the performance of the INPADR. Meanwhile, we plan to integrate more biological data to measure ADR similarity and protein similarity more accurately in future work.

## Additional Information

**How to cite this article**: Chen, X. *et al.* Large-scale identification of adverse drug reaction-related proteins through a random walk model. *Sci. Rep.* **6**, 36325; doi: 10.1038/srep36325 (2016).

**Publisher’s note:** Springer Nature remains neutral with regard to jurisdictional claims in published maps and institutional affiliations.

## Supplementary Material

Supplementary Information

Supplementary Dataset 1

## Figures and Tables

**Table 1:** The topological properties of the protein-ADR network.

Properties	Values	Properties	Values
Number of nodes	411	Shortest paths	87,242
Number of edges	503	Characteristic path length	5.607
Cluster coefficient	0	Density	0.006
Connected components	37	Average number of neighbors	2.448
Diameter	15	Network heterogeneity	1.714
Radius	1	Centralization	0.121
