# Supplementary material for: Large-scale identification of adverse drug reaction-related proteins through a random walk model
Source: Sci Rep. 2016 Nov 2;6:36325. doi: 10.1038/srep36325 (PMC5090865; doi:10.1038/srep36325)
Supplement: Supplementary Information [file srep36325-s1.pdf]

Supporting Information

Large-scale identification of adverse drug reaction-related proteins through a random walk model

Xiaowen Chen[1,+], Hongbo Shi[1,+], Feng Yang[1], Lei Yang[1], Yingli Lv[1], Shuyuan Wang[1], Enyu Dai[1], Dianjun Sun[2,*], Wei Jiang[1,*]

[1]College of Bioinformatics Science and Technology, Harbin Medical University, Harbin 150081, China

[2]Center for Endemic Disease Control, Chinese Center for Disease Control and Prevention, Harbin Medical University, Harbin 150081, China

*****corresponding author: hrbmusdj@163.com and jiangwei@hrbmu.edu.cn

[+] these authors contributed equally to this work

Supporting Information

Supplementary Table S1. The top 50 predictive proteins for each ADR.

Supplementary Table S2. The biological processes validated in the literature for hypertension and atherosclerosis.

Supplementary Figure S1. The biological characteristics of the potential proteins related to atherosclerosis.

Supplementary Table S2. The biological processes validated in the literature for hypertension and atherosclerosis.

ADR	The biological process validated in the literature
Hypertension	1. adenylate cyclase-inhibiting serotonin receptor signaling pathway (GO:0007198)<br>2. baroreceptor response to decreased systemic arterial blood pressure (GO:0001982)<br>3. CD8-positive, alpha-beta T cell differentiation involved in immune response (GO:0002302)<br>4. cellular response to L-ascorbic acid (GO:0071298)<br>5. cellular response to mineralocorticoid stimulus (GO:0071389)<br>6. cellular response to vitamin E (GO:0071306)<br>7. glycine import (GO:0036233)<br>8. interleukin-15-mediated signaling pathway (GO:0035723)<br>9. interleukin-8-mediated signaling pathway (GO:0038112)<br>10. lipoprotein lipid oxidation (GO:0034439)<br>11. negative regulation of branching involved in lung morphogenesis (GO:0061048)<br>12. negative regulation of collagen biosynthetic process (GO:0032966)<br>13. negative regulation of gap junction assembly (GO:1903597)<br>14. negative regulation of glutamine transport (GO:2000486)<br>15. negative regulation of heart rate involved in baroreceptor response to increased systemic arterial blood pressure (GO:0001985)<br>16. negative regulation of monocyte extravasation (GO:2000438)<br>17. negative regulation of pancreatic stellate cell proliferation (GO:2000230)<br>18. negative regulation of phospholipid efflux (GO:1902999)<br>19. negative regulation of plasma membrane long-chain fatty acid transport (GO:0010748)<br>20. negative regulation of renin secretion into blood stream (GO:1900134)<br>21. negative regulation of the force of heart contraction involved in baroreceptor response to increased systemic arterial blood pressure (GO:0001986)<br>22. neutrophil apoptotic process (GO:0001781)<br>23. norepinephrine-epinephrine vasoconstriction involved in regulation of systemic arterial blood pressure (GO:0001994)<br>24. phospholipase D-activating G-protein coupled receptor signaling pathway (GO:0031583)<br>25. positive regulation of chronic inflammatory response to antigenic stimulus (GO:0002876)<br>26. positive regulation of dendritic spine maintenance (GO:1902952)<br>27. positive regulation of hepatic stellate cell activation (GO:2000491)<br>28. positive regulation of phospholipid efflux (GO:1902995)<br>29. positive regulation of STAT protein import into nucleus (GO:2000366)<br>30. positive regulation of vitamin D biosynthetic process (GO:0060557)<br>31. protein kinase C deactivation (GO:0042313)<br>32. regulation of blood volume by renal aldosterone (GO:0002017)<br>33. regulation of carbohydrate phosphatase activity (GO:0032113)<br>34. regulation of glutamine transport (GO:2000485)<br>35. regulation of lipid transport across blood brain barrier (GO:1903000)<br>36. regulation of lipoprotein lipid oxidation (GO:0060587)<br>37. regulation of monocyte extravasation (GO:2000437)<br>38. regulation of neuronal action potential (GO:0098908)

	39. regulation of STAT protein import into nucleus (GO:2000364)<br>40. response to prostaglandin F (GO:0034696)<br>41. response to vitamin B1 (GO:0010266)<br>42. smooth endoplasmic reticulum calcium ion homeostasis (GO:0051563)
Atherosclerosis	1. activation of phospholipase A2 activity by calcium-mediated signaling (GO:0043006)<br>2. active induction of host immune response by virus (GO:0046732)<br>3. cellular response to potassium ion starvation (GO:0051365)<br>4. cellular response to vitamin E (GO:0071306)<br>5. helper T cell extravasation (GO:0035684)<br>6. lipoprotein lipid oxidation (GO:0034439)<br>7. MAPK import into nucleus (GO:0000189)<br>8. mineralocorticoid receptor signaling pathway (GO:0031959)<br>9. negative regulation of natural killer cell chemotaxis (GO:2000502)<br>10. negative regulation of pancreatic stellate cell proliferation (GO:2000230)<br>11. negative regulation of phospholipid efflux (GO:1902999)<br>12. negative regulation of phospholipid transport (GO:2001139)<br>13. negative regulation of smooth muscle cell-matrix adhesion (GO:2000098)<br>14. negative regulation of transcription from RNA polymerase II promoter in response to endoplasmic reticulum stress (GO:1990441)<br>15. negative regulation of vascular wound healing (GO:0061044)<br>16. phospholipase D-activating G-protein coupled receptor signaling pathway (GO:0031583)<br>17. positive regulation of calcidiol 1-monooxygenase activity (GO:0060559)<br>18. positive regulation of chronic inflammatory response to antigenic stimulus (GO:0002876)<br>19. positive regulation of hepatic stellate cell activation (GO:2000491)<br>20. positive regulation of leukotriene production involved in inflammatory response (GO:0035491)<br>21. positive regulation of low-density lipoprotein particle receptor catabolic process (GO:0032805)<br>22. positive regulation of myosin light chain kinase activity (GO:0035505)<br>23. positive regulation of NAD+ ADP-ribosyltransferase activity (GO:1901666)<br>24. positive regulation of phospholipase C-activating G-protein coupled receptor signaling pathway (GO:1900738)<br>25. positive regulation of phospholipid efflux (GO:1902995)<br>26. positive regulation of STAT protein import into nucleus (GO:2000366)<br>27. protein kinase C deactivation (GO:0042313)<br>28. Regulation of blood coagulation, intrinsic pathway (GO:2000266)<br>29. regulation of leukotriene production involved in inflammatory response (GO:0035490)<br>30. regulation of lipoprotein lipid oxidation (GO:0060587)<br>31. regulation of myosin light chain kinase activity (GO:0035504)<br>32. regulation of phospholipase C-activating G-protein coupled receptor signaling pathway (GO:1900736)<br>33. regulation of phospholipid efflux (GO:1902994)<br>34. regulation of smooth muscle cell-matrix adhesion (GO:2000097)<br>35. regulation of STAT protein import into nucleus (GO:2000364)

	36. response to cobalamin (GO:0033590)<br>37. smooth endoplasmic reticulum calcium ion homeostasis (GO:0051563)

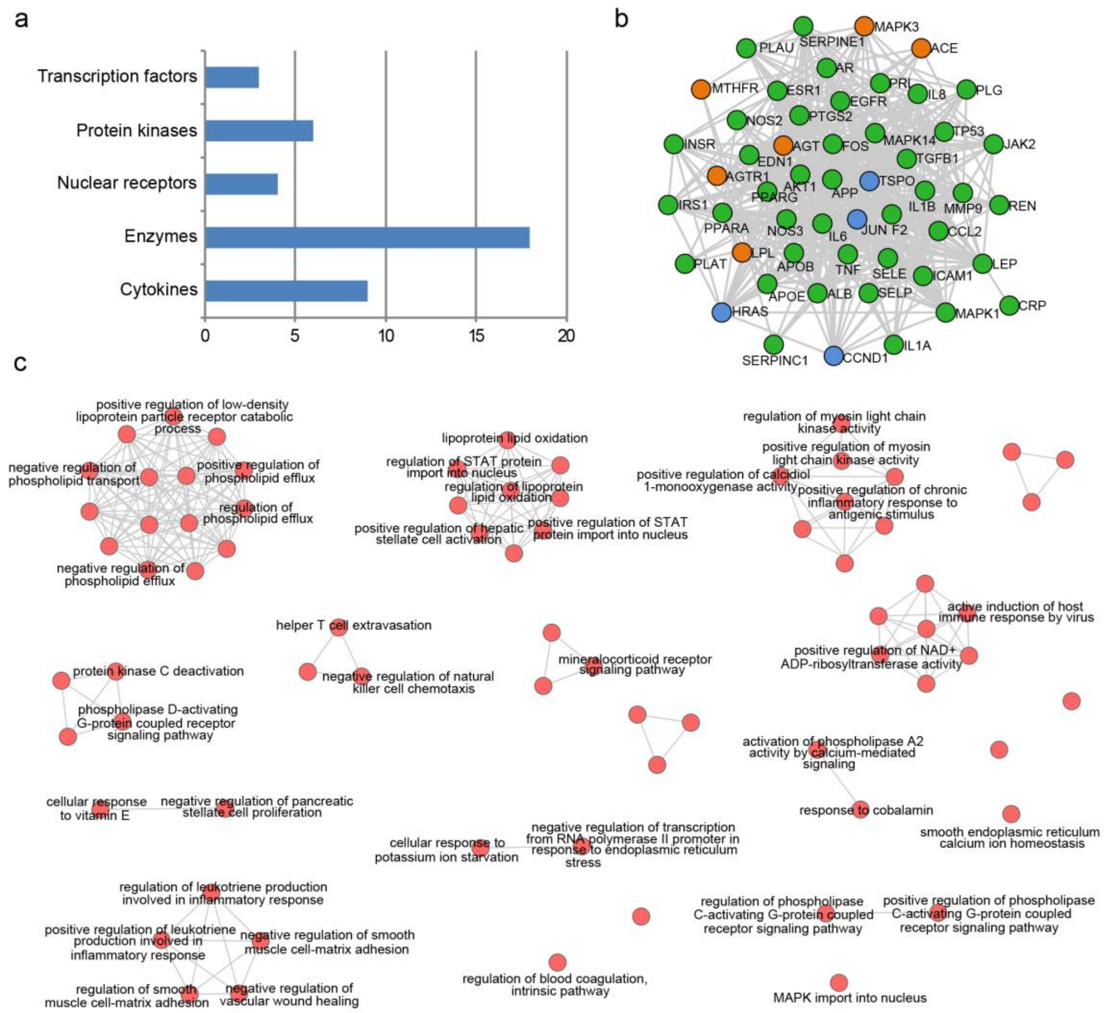

Supplementary Figure S1. The biological characteristics of the potential proteins related to atherosclerosis. (a) KEGG classification of the top 50 potential proteins related to atherosclerosis. (b) The interactions between known atherosclerosis-related proteins and the top 50 potential proteins. Orange nodes denote known seed atherosclerosis-related proteins. Green nodes denote validated top 50 potential proteins. Blue nodes denote unvalidated top 50 potential proteins. (c) A similar network of enriched GO biological processes by the top 50 potential proteins is built by the ClueGo. The biological processes are labeled, which are confirmed in the literature.
